# Evidencing the effectiveness of upper limb prostheses: a multi-stakeholder perspective on study requirements

**DOI:** 10.3389/frhs.2023.1213752

**Published:** 2023-12-21

**Authors:** Hannah Jones, Alix Chadwell, Matthew Dyson

**Affiliations:** ^1^ Intelligent Sensing Laboratory, School of Engineering, Newcastle University, Newcastle upon Tyne, United Kingdom; ^2^ Edinburgh Neuroprosthetics Laboratory, School of Informatics, University of Edinburgh, Edinburgh, United Kingdom; ^3^ Centre for Human Movement and Rehabilitation, University of Salford, Manchester, United Kingdom

**Keywords:** upper limb prosthetics, policy, stakeholder engagement, outcome measures, quality of life

## Abstract

The provision of upper limb prosthetic devices through the National Health Services (NHS) within the United Kingdom is driven by national policies. NHS England have recently published a new policy to provide multi-grip myoelectric hands. The policy highlighted that there was limited evidence to support its deployment and it will be reviewed should new information arise. The clear identification of the evidence gap provides an opportunity for the academic research community to conduct studies that will inform future iterations of this and other upper limb prosthetic related policies. This paper presents a summary of findings and recommendations based on two multi-stakeholder workshops held in June 2022 and July 2022, which explored the design requirements for policy-driven research studies. The workshops involved people from a broad range of stakeholder groups: policy, academia, NHS clinical and management, industry, and a person with upper limb absence. The workshop discussions focused on the research questions that NHS England identified in the policy evidence review: (1) Clinical Effectiveness; (2) Cost Effectiveness; (3) Safety; and (4) Patient Subgroups. The recommendations based on stakeholder discussions included the need to gather qualitative and quantitative research evidence, use goal-based outcome measures, and conduct longitudinal studies. Future research studies also need to address the complexities of conducting national and international policy-driven research, such as clinical resource capacity and participant involvement.

## 1. Introduction

Upper limb prosthetic policy in England is in a state of transition. For many years, the most advanced prosthetic hands available on the National Health Service (NHS) were limited to opening and closing in a pinch grip controlled by signals generated from a person's muscles, named as standard myoelectric. In September 2022, following an evidence review and needs assessment undertaken at a national level, NHS-England made the decision to routinely provide patients across England with more advanced prostheses called multi-grip myoelectric hands (1). This process of reviewing the needs, planning and prioritising funding, and subsequently monitoring and reviewing the clinical service, is known within the NHS as the commissioning process (2). During the commissioning process, the NHS Specialised Services Clinical Panel who review the documents, highlighted that, there is currently limited evidence on the clinical and cost effectiveness of multi-grip myoelectric hands, but that the policy proposition would address a gap in equity (3). The decision was therefore made to change the stance across the NHS to make multi-grip myoelectric hands routinely available. However, the Clinical Commissioning Policy stated that a review of the policy will be conducted when new information is received indicating that the policy requires revision (1). The associated Evidence Review states: “Further research, preferably involving the randomisation of participants to different groups, is required to further understand the clinical effectiveness, safety and cost effectiveness of myoelectric multi-grip prosthetics compared to standard prosthetics” (4). This current state-of-play presents a pertinent opportunity for the research community to design, develop, and conduct studies that aim to better inform this and other future upper limb prosthetics policies. This paper summarises findings from a consensus-based process aimed at improving the design of research studies, which, we believe is a first step towards addressing this evidence gap.

This consensus-based process aims to minimise long-term research costs and improve the quality of the research studies. Upper limb prosthesis provision is complex, the patient population is heterogenous, and rehabilitation goals vary by person. As such, a strong evidence-base involving the randomisation of participants would require large-scale research studies to be undertaken, however, due to the population size, access to a significant participant pool has historically been difficult. Studies often include small numbers of participants local to a research institution and vary widely in their approaches to evaluation. Furthermore, the provision of upper limb prostheses is costly, which also impacts upon the cost of running research studies that use advanced devices such as multi-grip myoelectric hands. It is therefore critical to gain a consensus on the study designs that would be suitable to generate the evidence required by policymakers and device funders prior to running studies.

This paper presents a summary of discussions from two multi-stakeholder workshops that highlighted areas of consideration when designing research studies to inform upper limb prostheses policies. The discussions were broadly based on the research questions that NHS England identified in the policy evidence review: (1) Clinical Effectiveness; (2) Cost Effectiveness; (3) Safety; and (4) Patient Subgroups (4).

## 2. Workshop design

Two multi-stakeholder workshops were held; one online in June 2022 and one in-person in July 2022, which had 14 and 24 people in attendance, respectively. Each workshop was held over the course of one day: online 5.5 h, in-person 6 h (the agendas are available in Supplementary Material A). The online workshop had shorter topic discussions to minimise fatigue during online interaction.

The recruitment for the workshops was conducted online via e-mail invitations to the steering group's professional networks (for example, through the International Society of Prosthetics and Orthotics UK), and advertisement with a workshop flyer on Twitter. The project steering group includes members from upper-limb prosthetics academic research groups across the UK and Ireland, policymakers, and leading charitable and professional organisations. Pre-read material was sent to all participants prior to the workshops, which outlined background information about current methods of measuring clinical effectiveness of upper limb prostheses. This material was sent to provide an opportunity for participants to become familiar with the terms of reference that would be used within the workshops.

The workshops involved adults (18 years or older) from the UK and Ireland: people with experience of policy, academia, NHS clinical and management, upper limb absence, and industry (Table 1), one of whom had lived experience of upper-limb absence and prosthesis use; please note that some attendees fell into multiple stakeholder groups. Workshop attendees were pre-allocated into groups that, where possible, had representation from each stakeholder group to enable a range of perspective to be discussed. The online workshop had 3 groups and the in-person workshop had 4 groups.

**Table 1:** Workshops 1 and 2 participant stakeholder groups.

Stakeholder group	Number of participants
People with upper limb absence	1
Policy	2
Academia	14
NHS Clinical and Management	22
Industry	2

The discussions at each workshop were facilitated by trained facilitators. The discussions were captured on post-it notes by both the attendees and the facilitators. During the online workshop each author took part in a different group and during the in-person workshop, two authors were facilitators, whilst the third moved between groups listening to an overview of the discussions across the room. This approach ensured that all the authors had a depth of knowledge of the content that was captured during both workshops to inform the analysis. Information captured from the online workshop discussions was documented on a digital whiteboard (Supplementary Material B). Information captured from the in-person workshop discussions were documented on paper-based worksheets (Supplementary Material C). Photographs of workshop content were captured to assist the analysis. Neither workshop was audio or video recorded.

The analysis of findings from both workshops was conducted by the authors based on a thematic approach (5) to identify main discussion areas and recommendations. Authors agreed on the thematic approach and each author independently analysed the findings within the agreed framework. Authors shared the analysis between themselves and identified common discussion areas and recommendations. The analysis was discussed collectively and reviewed based on the workshop findings. Authors addressed their own biases throughout this process by sharing findings with the wider project steering group, providing an opportunity for critique and discussion.

### 2.1. Workshop topics

The workshop discussion topics were designed by the authors with the support of 5 senior academics from other UK and Ireland universities who were involved in the wider project steering group. The starting point of identifying the workshop topics began with the research questions from the NHS England evidence review (4), which explore multi-grip myoelectric hands through the lens of: (1) Clinical Effectiveness; (2) Safety; (3) Cost Effectiveness; and (4) Patient Subgroups. The evidence review (4) also separates clinical effectiveness into critical and important outcomes. Under critical, they include functional outcome measures, activities of daily living, and quality of life; under important, they list prosthetic abandonment, patient satisfaction, prosthetic acceptability, device durability and frequency of replacement and/or re-fitting.

The authors and members of the steering group agreed that the research expertise in the UK and Ireland was likely to lean towards the assessment of Clinical Effectiveness, therefore this topic was given a higher time weighting in the workshops. It was also agreed that to effectively cover the critical outcomes, Clinical Effectiveness should be split into two sub-topics: (1) function and (2) lived experience (which encompasses topics such as quality of life). Safety, Cost Effectiveness and Patient Subgroups were also used as discussion topics within the workshops. In addition to the questions posed by NHS England, the lack of participant and clinician engagement with upper limb prosthetics research have been identified as hurdles to the success of policy-driven research studies. We therefore also asked the workshop participants to discuss methods of engaging people with these types of research studies.

### 2.2. Workshop questions

All groups were asked the same questions for each topic at both workshops (Supplementary Material B and C).

Clinical Effectiveness was the first topic at both workshops, where participants were asked to discuss how function and lived experience might be assessed to inform policy. Prompt questions for functional assessment included what outcome measures work, what does not work, what needs validation and what needs improvement? Prompt questions for lived experience assessment were around the challenges to the way lived experience is currently assessed. Both function and lived experience discussions within the Clinical Effectiveness topic included questions on identifying gaps and opportunities for assessment.

The second topic of the workshops explored which Patient Subgroups should be considered for research studies. In addition, to help guide the study designs, this session brought in a broader conversation around Patient Involvement and Engagement in research studies. The participants focused on aspects such as challenges and incentives, as well as how and where people could find out about getting involved in studies. This discussion topic also explored the importance of having clinical collaboration and input into research studies and what challenges currently exist in terms of the practicalities, such as resource capacity and time constraints.

The third and fourth topics of the workshops addressed the questions around Cost Effectiveness and Safety collectively. These were combined due to time constraints and an awareness that many aspects of these two areas of discussion were likely to be addressed at a manufacturer level rather than through the research studies which were to be designed as an outcome of this work. Prompts included: how is Cost Effectiveness evaluated; patient safety; patient comfort; risk of harm; and the regulation process involved in certifying medical devices, such as CE marking in Europe (Conformité Européenne).

Throughout the workshops there were opportunities for each group to give feedback on their main discussion points to the wider group.

Each workshop concluded with a consolidation activity where participants were provided with a policy impact matrix (Supplementary Material C). Reflecting on the discussions from the rest of the day, participants were asked “what research could generate evidence which would inform policy in the short, medium and long term?”. Groups were then asked to map these initial research study ideas onto a matrix where the perceived policy impact was mapped against the time taken to undertake the work.

The actionable recommendations within this paper are informed by the workshop discussions and this concluding activity from both workshops. The workshops were part of a patient and public involvement exercise to contribute to the design of research studies. Ethical approval to undertake these workshops was given by the Newcastle University Faculty of Science Agriculture and Engineering Ethics Committee (reference: 18659).

## 3. Summary of workshop discussions

### 3.1. Clinical effectiveness

This section summarises the discussions on two Clinical Effectiveness sub-topics: (1) function; and (2) lived experience. This section is based on the first research question posed by NHS England surrounding the Clinical Effectiveness of multi-grip myoelectric hands (4).

**Outcome Measures.** There was a general consensus from workshop participants that existing outcome measures do not provide a holistic view of the success of a prosthesis. When assessing prosthesis performance, it is important that measures enable a combination of quantitative and qualitative data to be captured, sourced from both patients and clinicians. Workshop discussions were centred on outcome measures that go beyond categorisation and measure a range of activities, including functional performance and lived experience.

There were several challenges outlined relating to current outcome measures. The main challenge was a lack of measures that are specifically developed for people with upper limb loss or absence. For example, clinicians highlighted that quality of life measures, such as EQ-5D, are used within upper limb prosthetics clinics, but were originally designed to assess the impact of disease and health at a more generic level (6). By conducting such measures, patients and clinicians may not have a comprehensive overview of the impact on quality of life. For example, a recent study found patients with multi-grip hands to rate their quality of life higher than the norm (7). Furthermore, with measures such as the EQ-5D, patients scores often plateau, especially after adjusting to limb absence.

It was noted that workshop participants shared that there is a general bias within the field towards quantitative metrics. However, a mixed methods approach that combines quantitative and qualitative metrics may be required due to the broad definition of a successful prescription. It was highlighted that such an approach needs to ensure that qualitative measures, such as observational techniques and open-questions that enable people to share their lived experience, are sufficiently objective to inform upper limb prosthetics policy at a national level. Studies that go beyond traditional clinical measures, and/or use qualitative data were identified as gaps when discussing the assessment of prosthesis functionality. Observational research, which may not be hypothesis-driven, was raised by workshop participants as an equivalent when discussing how to measure lived experience.

Two main areas of improvement for current outcome measures were raised by the workshop participants: (1) increase uniformity in how measures are conducted such that comparisons can be made across patients; and (2) enable assessment over longer periods of time whilst remaining realistic on what measures will be undertaken and who will conduct the required assessment(s). It is pertinent to highlight that the latter point is dependent upon the capacity of clinical staff to contribute to longitudinal assessments, as raised by clinicians during the workshops. In addition, three best practice approaches for measuring function were raised: (1) the use of life course measurement approaches and/or assessing specific life events (e.g., becoming a parent or starting a new job) during a patient pathway; (2) tailoring assessments dependent on the stage of a patient's amputation journey (e.g., initial period post amputation such as 1 and 3 month clinical reviews); and (3) iterative testing with regular follow-ups.

Goal setting was discussed as a tool to contribute towards assessing functionality and lived experience by workshop participants. This may often involve individual goals, and/or goals related to roles within a family that change over time. It was noted that a trained professional needs to assess each patients' goals, and manage unrealistic goals and expectations. Workshop participants shared that a balance must be maintained between self-development and achievement of goals, to reduce the likelihood that people change their lifestyle to achieve prosthesis related goals. Furthermore, measuring patient progress against their personal goals was proposed as a potential method of standardising experimental analysis across patients.

It was highlighted that a quantitative method for assessing and monitoring individual goals is required. Furthermore, a validation of the Patient-Specific Functional Scale (PSFS) for use in upper limb prosthetics is an area of improvement that needs to be addressed.

Longitudinal assessment of the rehabilitation journey. Overall, there was an opinion from workshop participants that a better understanding of the success of prosthetic interventions can be gained by conducting more regular or continual assessment of clinical effectiveness over longitudinal periods. Current measures represent patient data from a relatively narrow timeframe (e.g., within clinic appointments), whereas patients' experience of limb loss or absence may vary daily, and assessment goals can change over time. It was highlighted that although every patient journey is described as unique, there are a series of relatively fixed stages across patient groups, for example starting rehabilitation, ending rehabilitation, and returning to work or school. Workshop participants shared that the experience someone has at these time points can be instrumental in determining what may happen in terms of their future prosthesis usage. A clinician during a workshop shared that if a patient becomes depressed after leaving rehabilitation and does not have the option to access clinical support, they tend to reduce use or entirely reject their prosthesis. While this comment was based on clinical experience, the observation may in part be explained by maladaptive coping strategies when adjusting to limb loss (8). The main challenge that emerged from these discussions was how to allocate sufficient time and resources to conduct measurements at appropriate stages of a rehabilitation pathway, and how to identify what these timepoints may be. Furthermore, the variability between people and different age brackets across children, young people, and adult populations, makes quality of life quantification difficult to achieve.

The broader context of clinical effectiveness included participant discussions that explored how a variety of factors feed into defining or reflecting upon lived experience. For example, psychological factors should be a part of assessments and family members could provide a valuable source of additional information. This could lead to decisions that are informed by several factors, rather than solely on the functional performance of how someone uses a prosthesis.

### 3.2. Safety

This section summarises the workshop discussions on the Safety topic. This section is based on the second question posed by NHS England (4) exploring the safety of prosthetic devices. The NHS England evidence review highlighted that there was no evidence of the safety of a myoelectric controlled multi-grip upper limb prosthesis compared with standard upper limb prostheses or no prosthetic use (4).

The European legislation conformity confirmation process, known as CE marking was discussed by several workshop participants as prohibiting new components coming to market, due to the timescales for completing this process; however, it was noted by one workshop group that despite these limitations the process remains essential. Prosthetic hands are generally classed in the United Kingdom as Class 1 medical devices (9) requiring a UKCA (United Kingdom Conformity Assessed), CE or CE UKNI (United Kingdom Northern Ireland) mark. This means that manufacturers and healthcare establishments who supply them must follow the UK medical device regulations. The timescale of CE marking was identified as a barrier, as was the notion that the CE marking process may prevent new components reaching market. It was also noted that CE marked devices often reach the market with limited evidence of functionality and once modified, liability for the device lies with the prosthetic clinical rehabilitation service.

Training was raised in numerous domains by participants including training clinicians to inform and guide patients through the decision making process of prescribing a prosthesis; prosthetic device training to reduce abandonment; training in CE safety; patient education and ensuring prosthetics training includes modern socket design.

Physical and psychological patient comfort was raised during the workshop discussions, particularly by clinicians, academics and the person with limb absence. Patient comfort, in terms of the socket-patient interface and fit, was acknowledged as a common problem and an important overall outcome which receives limited research attention. Understanding current methods to reliably measure comfort was raised multiple times, as well as the need to develop new comfort metrics.

### 3.3. Cost effectiveness

This section summarises the workshop discussions on the Cost Effectiveness topic. This topic is the third question posed by NHS England (4). The NHS England evidence review highlighted that there was no evidence of the cost effectiveness of a myoelectric controlled multi-grip upper limb prosthetic compared with standard upper limb prostheses or no prosthetic use (4).

Clinical factors included quantifying the time, resources and expertise necessary to deliver a specific prosthesis and administer outcome measures required both by the health service and as part of research studies. There were also discussions from participants that stressed the importance of cost in clinical decision making. Participants highlighted how different centres may use different budgeting approaches as this will be guided by their local trust and this can factor into the treatment approach and the devices they prescribe to patients. Further the financial setup may differ across different clinical centres. For example, some prosthetists are employed directly by the NHS, and others may be employed by private companies that either deliver prosthetic services within the NHS or alternatively serve the private patient sector. This scenario factors into the ability to cost these roles in to grant applications, as different clinical centres may have varying budgeting structures. All of these factors can add complexity to research studies. Clinical stakeholders also highlighted the cost associated with purchasing and delivering outcome measure assessments, including the time taken to train clinicians.

Hidden costs in the provision of prostheses often fall into a separate budget line and may not be identified as part of the overall cost of a device. Therefore, there are currently challenges in how cost is measured. For example, a prosthesis that has been used for decades appears to be cost effective, but parts of the device may have been replaced multiple times on a separate budget line. This might include out of warranty replacements, repairs, wear-and-tear, and consumables such as replacement gloves. These costs, alongside costs related to device abandonment were all raised by participants as being rarely reviewed and/or having limited information available. The demand for consumables affects the often high spend on upper limb prosthetic devices. Replacement costs were identified by academic and clinical workshop participants as being particularly complex in paediatric prosthetics as children outgrow devices.

The long-term cost of prescribing a prosthesis was noted by participants as being difficult to track and measure. Two outline approaches were proposed: (1) evidencing long term achievements and contributions to society of people with upper limb absence; and (2) evidencing the long-term implications of not using an upper limb prosthesis. More specific measurements proposed included assessing: quality of life over a longitudinal period, patient income before and after prosthesis fit, rates of adverse events, and the impact on other healthcare services within the NHS, such as social services.

### 3.4. Patient subgroups

This section summaries the workshop discussions on the Patient Subgroup and Patient Involvement topics. The fourth research question posed by NHS England queried whether there were subgroups of patients who may benefit more from a multi-grip myoelectric upper limb prosthetic than the wider population of interest. The NHS England evidence review highlighted that there was no evidence to support this either way (4). Rather than directly addressing which subgroups of patients may experience additional benefits from a multi-grip myoelectric upper limb prosthesis, workshop questions explored what patient subgroups existed and should be considered when designing representative research studies.

A wide range of subgroups were identified by workshop participants. The diverse nature of patient demographics and geographical location were frequent topics. The remaining topics of conversations were summarised into two groups, those that were generally described as discrete categories or groups and continuous ranges that patients fall somewhere within.

Discrete categories of patients were discussed during both workshops by participants. Although age was considered continuous, young people were described according to distinct groups ranging from infants through to teenagers. Other discrete categories included the reason for a patient's limb absence, i.e., whether the individual had a congenital absence or had acquired limb loss; the number of limbs impacted and their laterality; the discrete level of limb absence (e.g., above/below elbow); whether or not surgical interventions such as osseointegration or muscle reinnervation had been performed; whether an individual was a prosthesis user or not; and whether people were engaged with the medical system or not. Individuals were also catergorized based on their local relationships, i.e., whether they had support from family, siblings or a carer, and these relations were also raised by workshop attendees as potential research participants who could provide insight into the quality of a patient's rehabilitation journey.

Continual ranges used to describe patients covered a range of factors. The overall length of the residual limb used for functional control of upper limb myoelectric devices was discussed as a continuum. Other ranges included the recency of a patients' limb absence and how long they had spent interacting with healthcare services. A range of factors related to individual patient's lifestyles were raised by workshop participants, which generally focused on patients' levels of physical activity, such as their involvement in sports, and in factors that may influence their levels of physical activity, such as their employment status and job role.

### 3.5. Stakeholder engagement

This section summarises the workshop discussions about participant and clinical engagement in policy-focussed upper limb prosthetics research studies.

**Engagement from a clinical perspective.** Participants highlighted that clinicians should be encouraged and supported to have an active role in research studies. Authorship opportunities and Continuing Professional Development hours were presented as potential incentives by workshop participants. Time and funding were key topics of discussion; it was noted by clinicians that administering a study using clinical hours is challenging and should not infringe on the delivery of services, such as patient appointments. It was also noted that friction can be generated when clinical resources are redirected to research. Dedicated funding for prosthetists, clinicians and innovation/Research and Development departments to support research were raised as a possible solution by NHS clinicians and management at the workshops. It was noted that clinicians and academics at the workshops shared that NHS ethics for multi-site studies will be multi-faceted, and capacity should be included to manage and co-ordinate the ethical approval process for such studies.

Engaging and involving a wider cross section of participants was raised as a challenge to research study recruitment by workshop attendees. People who are happy with their prosthesis, or people who do not use their prosthesis do not necessarily engage regularly with a prosthetics centre, which depending on the recruitment process can bias the participant pool. It was suggested that patient groups and patient advocates should be involved in participant recruitment. It was noted that workshop participants felt expert patient ambassadors are not necessarily always representative of the broader patient community, and it is important to recognise the value of having shared project goals between all stakeholders. Furthermore, academic stakeholders at the workshop stressed that the research community needs to establish how to involve people during the experimental design process in a way that does not bias a study if the same group of people become research participants during the study.

## 4. Actionable recommendations

**Figure 1:**
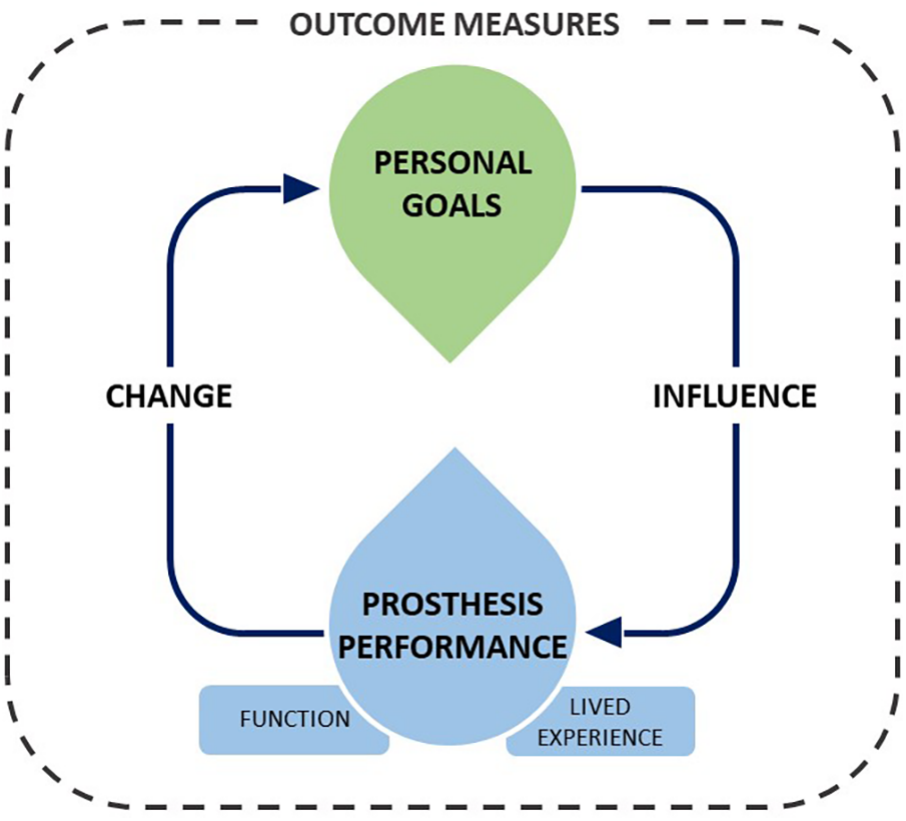
Representation of the dynamic relationship between outcome measures and prosthesis performance.

The following actionable recommendations are informed by the authors' summary of discussions from both workshops. The recommendations could span more than one of the commissioning research question areas (Clinical Effectiveness, Safety, Cost Effectiveness, and Patient Subgroups). The recommendations are not presented in priority order and may be applicable to both paediatric and adult population groups. Although the recommendations are presented under separate headings, crossovers can be identified, especially when considering the longitudinal pathway that people experience living with limb absence or limb loss. For example, outcome measures are informed by the identification and monitoring of personal goals, which influences prosthesis performance, which in turn change personal goals over time (Figure 1).

### 4.1. Gather qualitative and quantitative research evidence

Quantitative and qualitative research is required to generate evidence on the effectiveness of multi-grip myoelectric hands on both an individual and a national scale.

The implementation of qualitative techniques would allow for a deeper insight into patients' perspectives on how successful their prescription has been. It is worth highlighting that the NHS multi-grip myoelectric hand policy assigns 30% weighting for prosthetic provision on the patient experience view (1). It is recommended that an upper limb prosthesis specific quality of life measure is developed, which can be incorporated into mixed method studies that gather both quantitative and qualitative research findings.

### 4.2. Use of goal-based outcome measures

It was commonly agreed and highlighted by all groups that the best method to assess the effectiveness of prosthetic intervention would depend on individual personal goals. It was also highlighted that goals could change over time as people become more experienced and skilled with their prosthesis or as their personal circumstances change. Progression against personal goals was proposed more than once as a valuable outcome measure and it is recommended that this should be included in any research studies aiming to inform policy decisions. By doing so, outcomes could develop from the standard categorisation model towards incorporating qualitative data that demonstrates progress against personal goals. A suggestion was made that this could be a patient reported outcome measure, but this can be challenging to utilise on a broader scale when exploring the overall clinical effectiveness of an intervention. Alternative methods should therefore also be sought and where needed validated. Where possible, clinically meaningful difference values should be developed. In addition, methods for capturing individual progress and utilising this to inform the collective success of an intervention should be explored. It was noted that personal goals may change based on prosthesis performance (Figure 1) and that these changes may therefore highlight meaningful indicators of how a person perceives the functionality of their prosthesis.

### 4.3. Conduct longitudinal studies

Each experience of limb loss or absence is unique and lasts throughout a person's lifetime. During this normally multi-decade long experience, people may fluctuate in the level of health and care services they receive from upper limb prosthetic clinics. However, there can be common stages that more than one person experiences. For example, when somebody first has an amputation, there can be a set of fixed stages as part of an intensive clinical rehabilitation pathway. These pathways may take several months, if not years to navigate through, with prolonged periods of time at home or within the community and/or workplace or school. Over this course of time, peoples' goals and needs may change, alongside their prosthesis usage patterns. It is recommended to gather qualitative and quantitative research data over the course of a rehabilitation pathway; findings relating to the clinical and cost effectiveness can then inform commissioning policy. Such studies will involve remote prosthesis data collection and patient reported outcomes when patients return home and adapt to life within a community, and/or workplace or school.

### 4.4. Measure wider social costs

The complexity of assessing the wider societal cost of commissioning prosthetic devices requires assessment of multiple interacting cost centres. For this reason, research should investigate savings and expenditure in: the wider social care associated with prosthesis use; associated clinical centres which may also be utilised; any associated co-morbidities or physical activities related to prosthetics; and in more general long-term prosthesis use. To effectively direct investment, it would also be valuable to identify whether specific devices or outcome measures are appropriate for different patient subgroups such as paediatrics.

### 4.5. Establishing baseline data

There is no current database characterising the population of people living with limb difference in the UK. There is a need to access and coalesce existing retrospective data from siloed sources to understand the current population and their usage patterns. This work would include utilising existing national data sources, such as the National Congenital Anomaly and Rare Disease Registration Service (NCARDRS), and by sourcing data from individual organisations, such as prosthetics service centres. In addition to understanding the demographics of the UK's limb different population, there is a need to monitor the supply, repair, refit and changes to prosthesis provision such that current costs and clinical workload can be accurately measured. This information is particularly needed in paediatric upper limb prosthetics, which remains an underserved and understudied area.

### 4.6. Educate and train

It is recommended that research studies assess current methods and develop new training-based interventions to enhance clinical services, and patients' experience of using a prosthesis. Education and training should focus on a broad range of stakeholders to improve overall expertise. Education and training sessions for clinical teams across several rehabilitation centres may deliver a cost-effective method of understanding the existing skill base. Sustainable and cost-effective training, such as self-management courses, may address the learning requirements of patients and their close family and/or friends. This could facilitate patient engagement with their clinical pathway and potentially enhance the experience of using a prosthesis.

### 4.7. Conduct data logging

Many of the ideas for research studies shared during the consolidation activity involved the use of logging technology to record prosthesis usage data. There was a general view amongst workshop participants that prosthetics manufacturers log and retain data of how their devices are used, but choose not to publish it. However, there was limited consensus on how useful data solely logged from prostheses would be for understanding real-world use. Consequently, a common feature of these discussions was how to create a form of activity monitoring in real-world conditions, outside of the laboratory. In comparison to prosthesis logging systems, said activity monitoring systems would also acquire contextual information such that performance metrics can be derived. Additional research is required to ensure that these approaches, especially those involving bespoke systems, and any associated methods of acquiring real-world data are sufficiently robust for large scale data collection.

## 5. Discussion

This paper presents a summary of discussion points sourced from two multi-stakeholder workshops held in June 2022 and July 2022, which explored questions raised within the current NHS England commissioning policy for myoelectric multi-grip prosthetic hands (1, 4). The workshops involved people from a range of stakeholder groups: policy, academia, NHS clinical and management, industry, and a person with upper limb absence. These workshops formed part of the early stakeholder involvement aspect of a broader, open and collaborative, policy-led research study design project.

Understanding and quantifying a successful prosthetic prescription is complex (10). Consequently, identifying appropriate outcome measures to use is also complex. Clinicians and researchers who participated in the workshops were keen that outcome measures assessed the goals of the patient. If the measure of success is how quickly someone can move an object from one place to another, but their original goal was to have a prosthesis which allowed them to brush their hair, then the measure is not useful for that purpose. This is likely why some occupational therapists in the workshops were strong advocates of measures such as the Canadian Occupational Performance Measure (COPM) and Assessment of Capacity for Myoelectric Control (ACMC), which are bespoke to a person's goals. Although these measures are useful on an individualistic level, they can be harder to integrate into an overall assessment of the success of the technology. In addition, these types of measures can take a long time to administer, and within the NHS, clinicians are limited in the time they are able to spend with patients. It is therefore important that if outcome data is to be centrally captured as part of the standard rehabilitation pathway (as is recommended within the NHS multi-grip myoelectric hand policy), then this data must also contribute and inform clinical practice. When measuring an individual's goals, it should be remembered that these may be relatively short-term and goals can change over time. If COPM is only conducted once every 12 months, the results may not be representative of the person's experiences. Living with limb absence is present throughout an individual's life course, where their needs and requirements may change. This journey should be reflected in the design of any research studies measuring the effectiveness of prosthesis provision.

People with upper limb absence can experience an onset of multiple long-term physical and mental health conditions, such as chronic pain and depression (11, 12). These changes can have an impact on peoples’ health and overall quality of life (13). As these changes emerge, the impact of prescribing multi-grip myoelectric hands may positively or negatively affect the prosthesis user, and other health and care services, such as physiotherapy and mental health. Furthermore, such a prescription may impact upon the responsibilities and emotional burden of carers or family members, which may increase over time as multiple conditions emerge (13). This scenario necessitates multiple investigations into the cost effectiveness and the lived experience of prosthesis users and carers based on the prescription of multi-grip myoelectric prostheses over a longitudinal period, which is reflected in the recommendations outlined within this paper. For this to be realised, collaboration with multiple stakeholders must be conducted, including policy makers, academics, health economists, clinicians, and prosthesis users.

Conducting collaborative research can lead to impactful outcomes for health-related research (14). The National Institute of Health and Care Research, advocates the importance of involving patient and public stakeholders in research studies, in addition to the emerging initiative of community and public engagement (15). In the field of policy development, considering the views, opinions, and experiences of multiple stakeholders could lead to positive change (16). However, implementing collaborative research in practice is complex. During both workshops, there were several discussions about identifying what benefit patients would gain from being involved in research studies. The benefits of involving patients in research are highly documented, however there are emerging academic perspectives on the potential ethical implications of doing this in practice for health-related research (17). There is also the reality of the relatively slow pace of research progress, which can impact upon the experience of being involved in research, in terms of patient fulfilment and participant retention. It is therefore critical that research studies clearly set out realistic study aims and expectations when recruiting and involving patients (18). Collaboration between researchers, NHS rehabilitation centre managers and clinicians, and patients will be key to the success of patient recruitment and involvement initiatives. However, capacity and capability building will be a core component in conducting such an approach. For example, patient groups may need support in developing health literacy, clinicians may need access to usable datasets, and researchers may require a knowledge exchange platform that facilitates collaboration with participants. These areas of capacity and capability building have been highlighted in a recent World Health Organisation framework for engagement (19). Furthermore, the UK Government Policy Lab has established a range of collaborative methods, which have the potential to be applied to policy-driven research studies (20). The methods can be linked to the emergence of *Design for Policy* over the past decade, which has permeated across multiple policy sectors (21, 22). These maturing initiatives are particularly relevant for involving users in policy-driven research; to ensure that methods are used to involve people by collaborating towards a shared goal (23).

This paper presents a summary of the first step towards addressing a shared goal in generating a consensus-based process to designing policy-led research studies. Based on the workshop discussions, there are several underserved areas with limited academic literature that need to be addressed before this goal can be achieved from a clinical and academic perspective. The areas identified were myoelectric training, prosthesis and socket comfort, the design and testing of outcome measures, and using qualitative approaches as measures in upper-limb prosthetic research. These areas are difficult to address and require an extensive degree of testing with people with limb absence. In terms of training, there is limited scientific evidence for some existing methods. In particular, the relationship between training myoelectric control in isolation and improvements in functional prosthesis use remains contentious, and this is a complex area and is difficult to validate (24, 25, 26). Regarding the assessment of comfort, neither of the most applied scales, the “Socket Comfort Score” or the “Comprehensive Lower-Limb Amputee Socket Survey”, are validated for upper-limb use (27, 28). Furthermore, socket comfort relates to socket fit and therefore myoelectric prosthesis function (29). Thus, socket comfort cannot be assessed in isolation, and likely requires a holistic approach where comfort is evaluated alongside functional gains. The Upper Limb Prosthetic Outcome Measures (ULPOM) working group made significant steps forward in identifying the most appropriate outcome measures for upper limb devices (30, 31). Although ULPOM made recommendations, there is still limited academic or clinical consensus on the use of outcome measures.

### 5.1. Limitations and future study considerations

The content presented within this manuscript is based on a project that is in an early phase of development. The project is a relatively new field of policy-driven research for upper limb prosthetics being run in the context of NHS policy. The reader should be aware of both the wider context of this project, and potential limitations with respect to the workshops from which these recommendations were drawn.

One such consideration is that the content presented in this paper is primarily based on the views and opinions shared by professionals, rather than users of prosthetic devices. The workshops focused on early-stage discussions around the design of research studies based on questions and background information sourced from policy documentation (1–4). The second stage of the project, which is currently on-going, comprises tailored workshops that involve a larger cohort of people with upper limb absence (adults and children) and their family and/or support network. This approach may minimise potential power dynamics which could occur in a multi-stakeholder workshop with policymakers, researchers, clinicians, and industry. Potential study designs based on the recommendations presented within this manuscript will be shared during the second stage workshops to inform the development of this body of work. Furthermore, to enhance stakeholder engagement during these workshops, digital and health literacy is a consideration that must also be addressed via approaches such as online and paper-based visual mediums (e.g., project animations, scenario mapping, and comic-book style print-outs) to facilitate collaboration and communicate the project before, during, and after a workshop.

Another limitation is that due to rail strike action in the United Kingdom, it was not possible to conduct the first workshop in-person, as planned. For methodological consistency, ideally a series of workshops should be conducted in one format, i.e., all online or all in-person, unless a mixed methods approach is used. Due to the difference in workshop format, it was decided to not audio or video record either workshop, so that the analysis of notes from both workshops was consistent. Furthermore, due to the anonymity of data collection and the scope of the funded project, comparisons between stakeholder groups cannot be identified from the content presented within this manuscript. Conducting workshops is a valuable method to elevate a variety of stakeholder opinions and ideas. However, workshops are limited to time, and discussions require high levels of concentration for all involved. The approach also requires a significant amount of time from the workshop participants, which can limit who has the capacity to attend across all stakeholder groups. A suggestion for future studies would be to apply a mixed methods approach including workshops, surveys, and one-to-one interviews to provide people the opportunity to engage in a format that best suits their needs and schedule.

By taking a national approach to research (involving stakeholders from across the United Kingdom and Ireland), it will be possible to generate evidence on a larger scale than previously achievable and ensure methodological consensus. This collaborative approach to evidencing policy decisions could be beneficial for other rare medical conditions involving specialised technology-based interventions.
