# Supplementary material for: Evidencing the effectiveness of upper limb prostheses: a multi-stakeholder perspective on study requirements
Source: Front Health Serv. 2023 Dec 21;3:1213752. doi: 10.3389/frhs.2023.1213752 (PMC10768005; doi:10.3389/frhs.2023.1213752)

Exploring Upper Limb Prosthetics Policy

A UK & Ireland Research Approach

How might **function** be assessed to inform policy?

What works?
What does not work?
What needs validation?
What needs improvement?

What are the gaps? What are the Opportunities?

Notes / Parking Lot

How might lived experience be assessed to inform policy?

How is lived experience currently assessed?
What are the challenges of assessing lived experience?

What are the gaps? What are the Opportunities?

Notes / Parking Lot

Patient Subgroups and Engagement

What patient subgroups should be considered for studies that aim to inform policy?

How might people be involved and engage with research studies that aim to inform policy?

What are the gaps? What are the Opportunities?

Notes / Parking Lot

How might cost effectiveness and safety be assessed in research?

How is cost effectiveness evaluated?
Patient Safety
Patient Comfort
Risk of Harm
CE Marking Process

What are the gaps? What are the Opportunities?

Notes / Parking Lot

Consolidation

What research could generate evidence which would inform policy in the short, medium, and long term?

Based on the previous 4 sessions: Place research questions / ideas / study designs on to the time vs impact matrix.

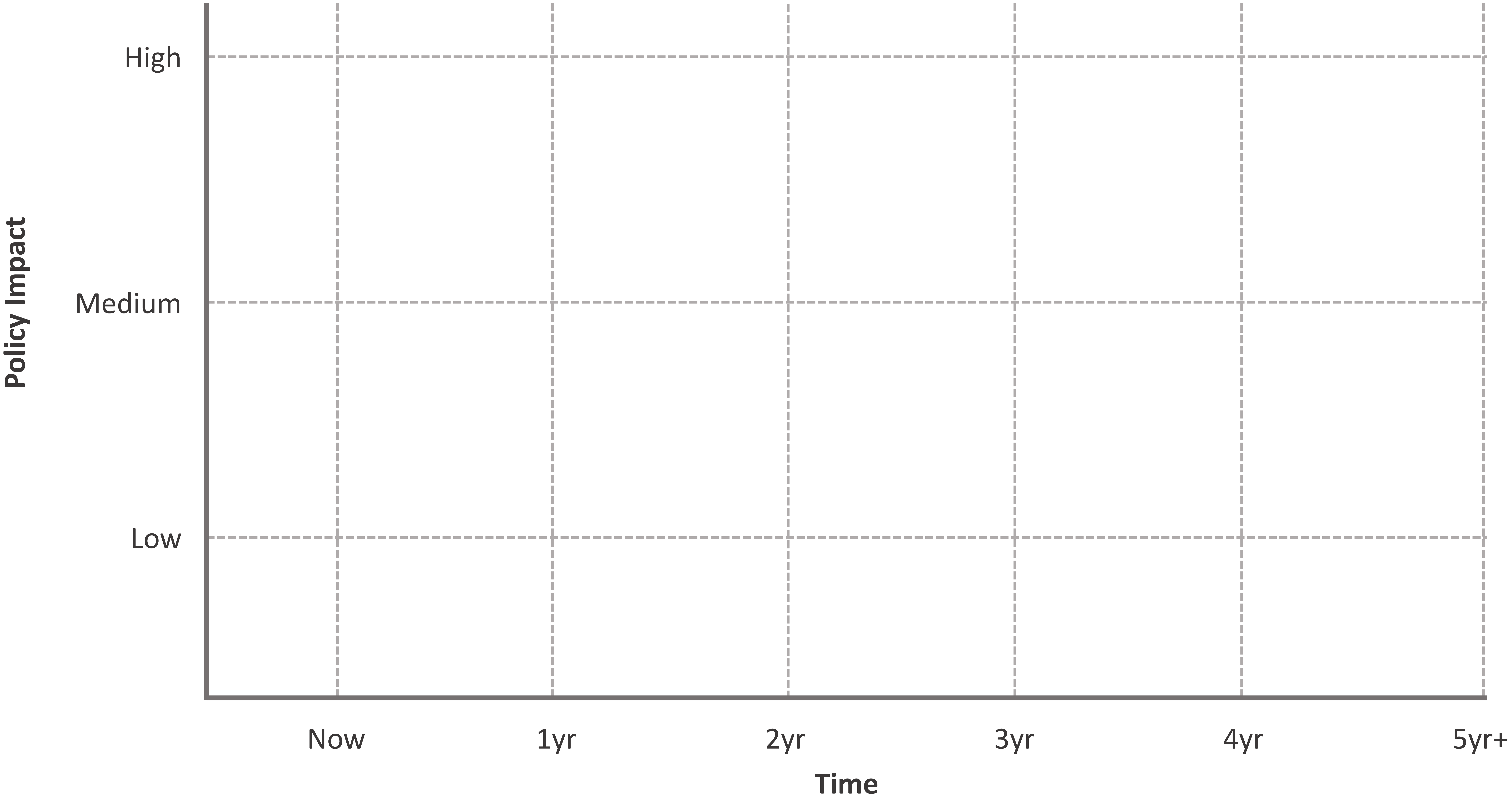

Supplement: Supplementary file 2 [file Datasheet2.pdf]
