# Supplementary material for: Evidencing the effectiveness of upper limb prostheses: a multi-stakeholder perspective on study requirements
Source: Front Health Serv. 2023 Dec 21;3:1213752. doi: 10.3389/frhs.2023.1213752 (PMC10768005; doi:10.3389/frhs.2023.1213752)
Supplement: Supplementary file 3 [file Datasheet3.pdf]

Exploring upper limb prosthetics policy

A UK & Ireland Research Approach

Newcastle University

Agenda | June 2022 | **Online** | 10:00 – 15:30 UK Time

09:50 **Sign-in via Zoom**

10:00 **Welcome and Introductions**

10:30 **Breakout Session 1: Function (Clinical Effectiveness)**

11:00 Break

11:10 **Breakout Session 2: Lived Experience (Clinical Effectiveness)**

Breakout Feedback: Summary of morning sessions

11:50 Lunch

13:00 **Afternoon Overview**

Breakout Session 3: Patient Subgroups and Engagement

13:40 Break

13:50 **Breakout Session 4: Cost Effectiveness and Safety**

14:20 Break

14:30 **Breakout Session 5 - Consolidation**

15:00 **Breakout Feedback**

15:15 **Workshop Summary and Next Steps**

15:30 **Workshop Close**

Exploring upper limb prosthetics policy

A UK & Ireland Research Approach

Agenda | July 2022 | **In-Person** | 10:00 – 16:00 UK Time

**10:00**	**Welcome and Introductions**
**10:30**	**Group Session 1: Function (Clinical Effectiveness)**
11:15	Break
**11:35**	**Group Session 2: Lived Experience (Clinical Effectiveness)**
	**Group Feedback: Summary of morning sessions**
12:10	Lunch

**13:00**	**Afternoon Overview**
	**Group Session 3: Patient Subgroups and Engagement**
**13:30**	**Group Session 4: Cost Effectiveness and Safety**
14:00	Break
**14:20**	**Group Session 5: Consolidation**
**15:00**	**Group Feedback**
**15:30**	**Workshop Summary and Next Steps**
**16:00**	**Workshop Close**
