# Supplementary material for: Evidencing the effectiveness of upper limb prostheses: a multi-stakeholder perspective on study requirements
Source: Front Health Serv. 2023 Dec 21;3:1213752. doi: 10.3389/frhs.2023.1213752 (PMC10768005; doi:10.3389/frhs.2023.1213752)
Supplement: Supplementary file 1 [file Datasheet1.pdf]

Exploring Upper Limb Prosthetics Policy

A UK and Ireland Research Approach

Blue Group

1. Function

How might function be assessed to inform policy?

What works?

What needs validation?

Outcome Measures

What doesn't work?

What needs improvement?

Where are the gaps? What are the opportunities?

Notes / Parking Lot

2. Lived Experience

How might lived experience be assessed to inform policy?

What are the current challenges of how lived experience is assessed?

Where are the gaps? What are the opportunities?

Notes / Parking Lot

3. Patient Subgroups and Engagement

What Patient Subgroups should be considered?

Adult
Pediatrics
Congenital
Acquired

How might people be involved and engage with research studies that aim to inform policy?

Where?
How?
Challenges?
Incentives?
Participants?
Clinical Centres?

Notes / Parking Lot

4. Cost Effectiveness and Safety

How might cost effectiveness and safety by assessed in research?

How is cost effectiveness evaluated?
Patient Safety
Patient Comfort
Risk of Harm
CE Marking Process

Notes / Parking Lot

5. Consolidation

What research could generate evidence which would inform policy in the short, medium, and long term?

Based on the previous 4 sessions:
Place research questions / ideas / study designs on to the time vs impact matrix.

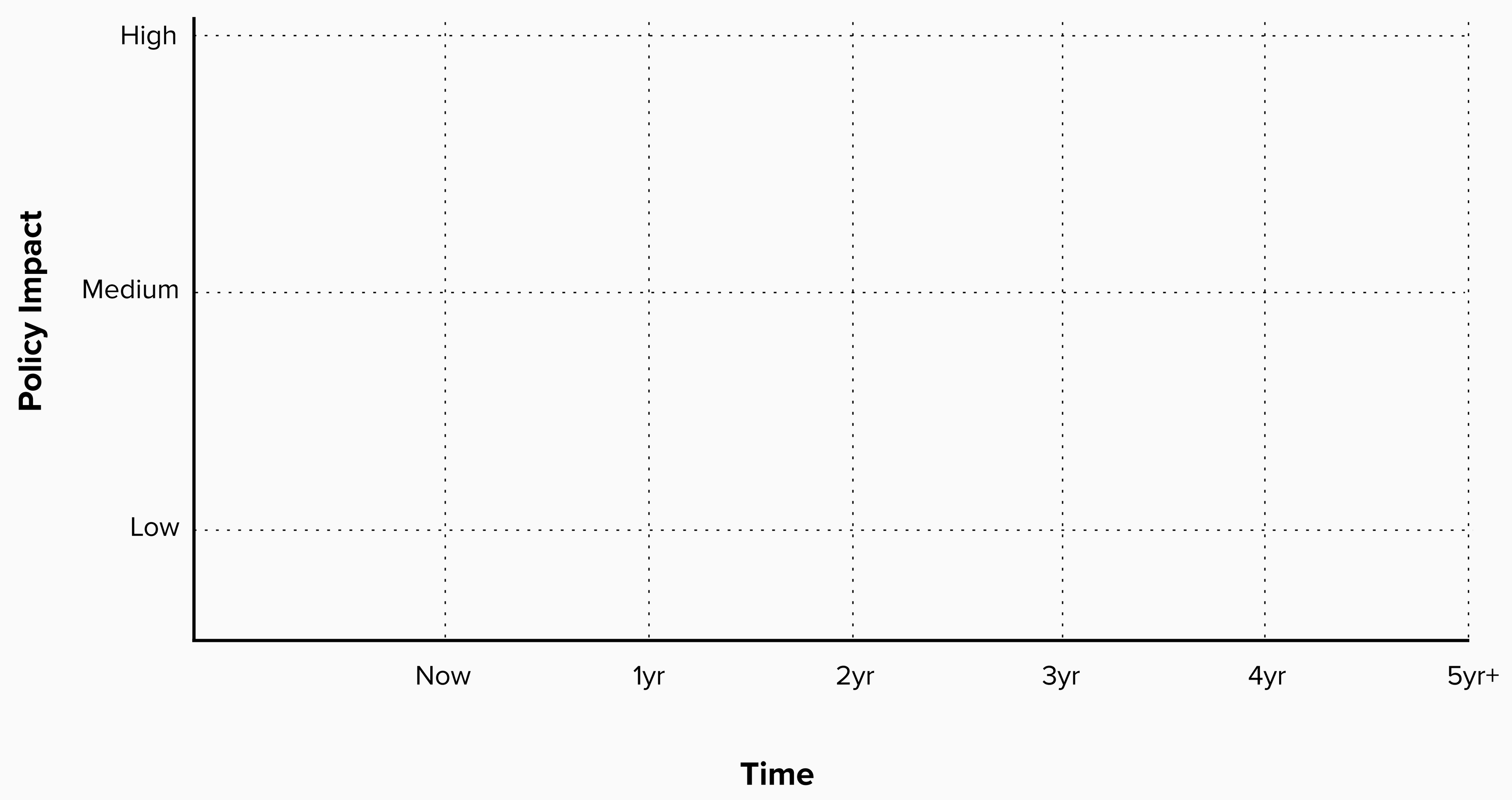

General Notes / Parking Lot
